# Inhibition of Toll-Like Receptor Signaling as a Promising Therapy for Inflammatory Diseases: A Journey from Molecular to Nano Therapeutics

**DOI:** 10.3389/fphys.2017.00508

**Published:** 2017-07-19

**Authors:** Wei Gao, Ye Xiong, Qiang Li, Hong Yang

**Affiliations:** ^1^Department of Respiratory Medicine, Shanghai First People's Hospital, Shanghai Jiaotong University School of Medicine Shanghai, China; ^2^Department of Respiratory Medicine, Changhai Hospital, Second Military Medical University Shanghai, China

**Keywords:** toll-like receptor (TLR), autoimmune diseases, inflammatory diseases, inflammation, TLR antagonist, TLR inhibitor, nanotherapeutics

## Abstract

The recognition of invading pathogens and endogenous molecules from damaged tissues by toll-like receptors (TLRs) triggers protective self-defense mechanisms. However, excessive TLR activation disrupts the immune homeostasis by sustained pro-inflammatory cytokines and chemokines production and consequently contributes to the development of many inflammatory and autoimmune diseases, such as systemic lupus erythematosus (SLE), infection-associated sepsis, atherosclerosis, and asthma. Therefore, inhibitors/antagonists targeting TLR signals may be beneficial to treat these disorders. In this article, we first briefly summarize the pathophysiological role of TLRs in the inflammatory diseases. We then focus on reviewing the current knowledge in both preclinical and clinical studies of various TLR antagonists/inhibitors for the prevention and treatment of inflammatory diseases. These compounds range from conventional small molecules to therapeutic biologics and nanodevices. In particular, nanodevices are emerging as a new class of potent TLR inhibitors for their unique properties in desired bio-distribution, sustained circulation, and preferred pharmacodynamic and pharmacokinetic profiles. More interestingly, the inhibitory activity of these nanodevices can be regulated through precise nano-functionalization, making them the next generation therapeutics or “nano-drugs.” Although, significant efforts have been made in developing different kinds of new TLR inhibitors/antagonists, only limited numbers of them have undergone clinical trials, and none have been approved for clinical uses to date. Nevertheless, these findings and continuous studies of TLR inhibition highlight the pharmacological regulation of TLR signaling, especially on multiple TLR pathways, as future promising therapeutic strategy for various inflammatory and autoimmune diseases.

## Introduction

**Figure 1:**
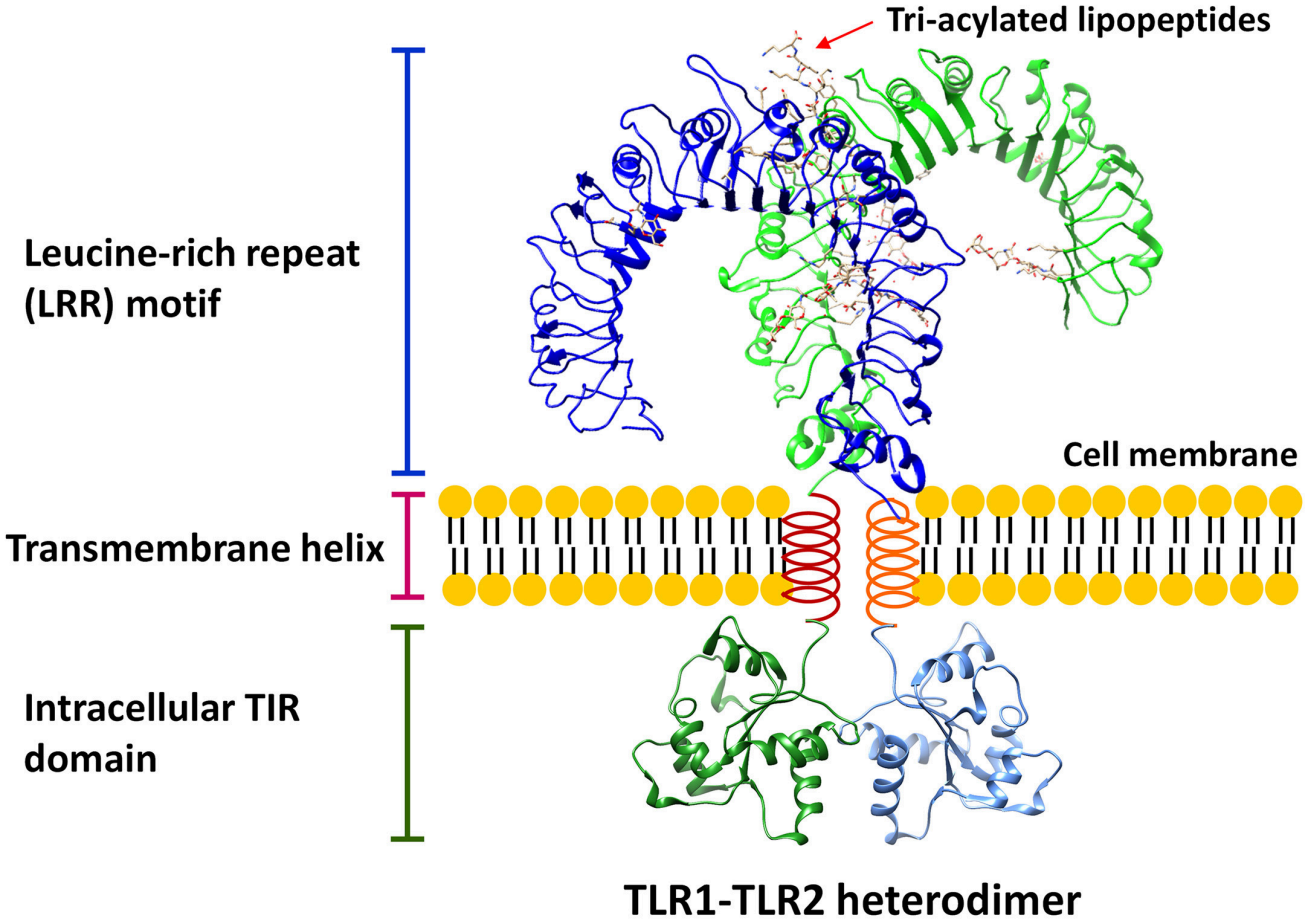
A representative structure of TLR. The conserved structural features of all TLRs consist of three critical components: (1) leucine-rich repeat (LRR) motif; (2) transmembrane helix; (3) intracellular TIR domain. The LRR structure is based on the model of TLR1-TLR2 heterodimer (Protein Data Bank, PDB, ID: 2z7x) interacting with 6 tri-acylated lipopeptides, Pam3CSK4, whereas the TIR domain homology model is based on TLR2 TIR structure (PDB ID: 1fyw).

Toll-like receptors (TLRs) are a family of pattern recognition receptors (PRRs) that can recognize and respond to a unique repertoire of distinct molecules referred to as pathogen-associated molecular patterns (PAMPs) and danger-associated molecular patterns (DAMPs; Kawai and Akira, 2010; Moresco et al., 2011). These conserved receptors belong to type I transmembrane glycoproteins and are made of three structurally important components: (1) the leucine-rich repeat motifs for ligand recognition at N-terminus; (2) a single transmembrane helix; and (3) a conserved cytoplasmic Toll/Interleukin-1 (IL-1) receptor (TIR) domain at C-terminus for intracellular signaling transduction (Botos et al., 2011; Bryant et al., 2015). A representative TLR structure is shown in Figure 1. Currently, a total of 13 TLRs have been identified, among which TLRs 1–10 are expressed in human despite the function of TLR10 is still unclear (Moresco et al., 2011). The expression of TLRs can be found in a variety of immune cells (e.g., dendritic cells, monocytes, macrophages, and B lymphocytes) and non-immune cells (e.g., epithelial cells, endothelial cells, and fibroblasts; Takeda et al., 2003). While TLR1, TLR2, and TLR4–6 are mainly found on the cell surface, TLR3 and TLR7–9 are primarily expressed in the endosomes (Gay et al., 2014). Two TLRs form a hetero- or homo-dimer that allow them to recognize and bind to different molecular patterns (Gay et al., 2014).

When engaging with PAMPs or DAMPs, TLRs can transduce the signaling to initiate innate and adaptive immune responses (Akira and Takeda, 2004; Palm and Medzhitov, 2009). The molecular pathways of TLR signal transduction can be simply categorized into two cascades (Akira and Takeda, 2004; Kawasaki and Kawai, 2014). One is through the main adaptor protein, myeloid differentiation factor 88 (MyD88). The ligation of TLR ligands results in the recruitment of MyD88 to the TIR domain and subsequently leads to activation of nuclear factor kappa-light-chain-enhancer of activated B cells (NF-κB) and mitogen-associated protein kinase (MAPK), which drive the expression of pro-inflammatory genes. The other is through the adaptor TIR-domain-containing adaptor-inducing interferon-β (TRIF), also known as the MyD88-independent pathway, to activate interferon (IFN) regulatory factors (IRFs) and NF-κB for the production of type I IFNs. Among all human TLRs, TLR3 exclusively signals through TRIF-dependent pathway while others use MyD88-dependent pathway; (Uematsu and Akira, 2007) notably, TLR4 can signal through both pathways in a time-dependent manner (Sato et al., 2003; Yamamoto et al., 2003).

TLR activation is one of the first defensive mechanisms utilized by the host to mount innate and subsequent adaptive immune responses to fight the invading pathogens and repair the damaged tissues (Takeda and Akira, 2005; Beutler, 2009; Palm and Medzhitov, 2009). However, dysregulated TLR signaling could disrupt the immune homeostasis (i.e., a self-regulating process to remain physiological balance) in the host by sustained pro-inflammatory cytokines and chemokines secretion. This often contributes to the development of many inflammatory and autoimmune diseases, such as systemic lupus erythematosus (SLE), sepsis, atherosclerosis, and asthma (Marshak-Rothstein, 2006; Tsujimoto et al., 2008; Drexler and Foxwell, 2010). Considering the pathological role of TLRs in these inflammatory diseases, inhibitors targeting TLR signaling might be beneficial in treating these disorders (Hennessy et al., 2010).

Despite many efforts have been put in developing (bio)molecular inhibitors targeting TLR signaling pathways, unfortunately, very few compounds are currently available for clinical uses. Therefore, it is important to search for novel TLR inhibitors that can be utilized both as therapeutic agents and as experimental reagents for dissecting innate immune molecular pathways. Manipulation of immune responsiveness using nanodevices provides a new strategy to treat human diseases, because nanotechnology offers many beneficial features for therapeutics development, including cell penetrating and targeting capabilities, desired biodistribution, improved pharmacokinetics, enhanced therapeutic activity, better biocompatibility, and *in vivo* tracking (bioimaging). Accordingly, novel nanodevices have emerged with bio-activities to potently regulate TLR signaling, aiming for inhibiting disease-associated TLR activation.

In this review, we first briefly summarize the pathophysiological role of TLRs in many diseases of acute and chronic inflammation as well as autoimmunity. These diseases include SLE in autoimmunity, infection-induced sepsis for acute inflammation, vascular system disorders across both acute and chronic inflammation, and other inflammation-associated diseases such as psoriasis, gastrointestinal cancer, asthma, and chronic obstructive pulmonary disease (COPD; Table 1). We then focus on the current knowledge in developing TLR antagonists and inhibitors at various stages from pre-clinical evaluation to clinical trials, including the most recent development of nano-based modulators on inhibition of TLR signaling, and discuss their potential uses in treating various inflammatory diseases. Particularly, we describe the design of these nanodevices and discuss their mechanism(s) of action in TLR inhibition. Due to the unique feature of nanodevices, these nano-inhibitors hold great promises in treating various inflammatory and autoimmune diseases.

**Table 1:** The role of TLR activation in the pathogenesis of inflammatory diseases.

**Types of TLR**	**Associated diseases**	**Evidence**	**References**
TLR2	SLE	High mRNA expression in patients; TLR2^−/−^ reduces autoantibody production	Komatsuda et al., 2008; Lartigue et al., 2009
	Sepsis	Up-regulated expression in patients; TLR2^−/−^ increases survival under microbial infection	Harter et al., 2004; Bergt et al., 2013
	Atherosclerosis	TLR2^−/−^ decreases vasculature inflammation	Mullick et al., 2005, 2008
	Hypertension	Renal IR injury	Khan et al., 2012
	Psoriasis	Respond to HSP60	Seung et al., 2007
	COPD	Cigarette smoke-induced inflammation; exacerbation under infection	Droemann et al., 2005; Tokairin et al., 2008
TLR3	Sepsis	TLR3^−/−^ is protective against microbial challenge	Cavassani et al., 2008
	Atherosclerosis	Promote plague instability	Ishibashi et al., 2013
TLR4	SLE	High mRNA expression in patients; TLR4^−/−^ reduces autoantibody production; elevated TLR4 presents SLE-like phenotype	Liu et al., 2006; Komatsuda et al., 2008; Lartigue et al., 2009
	Sepsis	Up-regulated expression in patients; TLR4^−/−^ is resistant to Gram-negative bacterial induced sepsis	Harter et al., 2004; Roger et al., 2009
	Atherosclerosis	TLR4^−/−^ decreases vasculature inflammation	Michelsen et al., 2004; Higashimori et al., 2011
	Hypertension	Respond to angiotensin II	Hernanz et al., 2015
	Psoriasis	Respond to HSP60	Seung et al., 2007
	IBD, gastrointestinal malignancies	Respond to resident microbes	Fukata et al., 2007; Fukata and Abreu, 2008
	Asthma	Respond to airway allergens; Th1/Th2 immune homeostasis	Dong et al., 2009
	COPD	Cigarette smoke-induced inflammation; exacerbation under infection	Karimi et al., 2006; Tokairin et al., 2008
TLR5	Psoriasis	Respond to TGF-α	Miller et al., 2005
TLR7	SLE	Up-regulated expression in patients; TLR7^−/−^ reduces autoantibody, IL-6 and IFN-α production	Christensen et al., 2006; Pisitkun et al., 2006; Subramanian et al., 2006; Lee et al., 2008; Kono et al., 2009
	Stroke	Dual role: protective in preconditioning, damaging in post-ischemia phase	Brea et al., 2011; Leung et al., 2012
	Psoriasis	Aggravate disease symptoms	Gilliet et al., 2004
TLR9	SLE	Regulatory role	Christensen et al., 2006; Nickerson et al., 2010; Chen et al., 2011; Jackson et al., 2014
	Sepsis	TLR9^−/−^ is protective against microbial challenge; TLR9 inhibition reduces disease severity	Yasuda et al., 2008; Liu et al., 2012; Hu et al., 2015
	Atherosclerosis	TLR9^−/−^ exacerbates atherosclerosis	Koulis et al., 2014
	Hypertension	Elevate blood pressure	McCarthy et al., 2015
	Psoriasis	Respond to TGF-α	Miller et al., 2005
	COPD	Cigarette smoke-induced inflammation	Mortaz et al., 2010

*SLE, systemic lupus erythematosus; IR, ischemia-reperfusion; HSP, heat shock protein; COPD, chronic obstructive pulmonary disease; IBD, inflammatory bowel diseases*.

## Toll-like receptor activation in autoimmune and inflammatory diseases

### Systemic lupus erythematosus (SLE)

Systemic lupus erythematosus (SLE) is a complex autoimmune disease characterized by the loss of tolerance to self-nuclear antigens and the production of autoantibodies, leading to the attack of the immune system on the self-components and healthy tissues. Recent evidences have suggested a close relationship between the endosomal TLR activation and the onset of SLE (Celhar and Fairhurst, 2014; Wu et al., 2015). It has been found that in B cells and plasmacytoid dendritic cells (pDCs), endosomal TLRs play an essential role in the generation of anti-nuclear antibodies and type I IFNs (Clancy et al., 2016). Particularly, in lupus-prone mouse models, TLR7 overexpression is associated with the production of autoantibodies and the development of autoimmune phenotypes (Pisitkun et al., 2006; Subramanian et al., 2006); on the other hand, in the situation of TLR7 deletion the levels of circulating autoantibodies and inflammatory cytokines, such as IL-6 and INF-α, are significantly decreased with improved disease symptoms (Christensen et al., 2006; Lee et al., 2008; Kono et al., 2009). In contrast to TLR7, the role of TLR9 in SLE is not well-defined. It has been shown that TLR9 is indispensable in the autoantibody production in B cells from several mouse studies (Christensen et al., 2006; Nickerson et al., 2010; Chen et al., 2011). However, the absence of TLR9 in these lupus-prone models does not help improve disease conditions, but leads to disease exacerbation (Christensen et al., 2006; Nickerson et al., 2010; Jackson et al., 2014). This suggests that TLR9 may have a regulatory role in the progression of SLE. Nevertheless, the expression of both TLR7 and TLR9 is up-regulated in the peripheral blood mononuclear cells (PBMC) from the SLE patients, and their levels are correlated with the production of IFN-α (Lyn-Cook et al., 2014), making them potential therapeutic targets of SLE.

In addition to endosomal TLRs, TLR2 and TLR4 have been found to participate in the pathogenesis of SLE based on the following evidences. First, their mRNA levels in PBMC of SLE patients are much higher than those in healthy controls (Komatsuda et al., 2008; Lee et al., 2016). Second, in a lupus mouse model, the deletion of TLR4 or TLR2 (to a lesser extent) decreases the levels of autoantibodies, and subsequently ameliorates the disease symptoms (Lartigue et al., 2009). Third, the elevation of TLR4 can induce lupus-like autoimmune disease (Liu et al., 2006). All these studies clearly demonstrated the importance of TLR signaling in SLE development and progression.

### Infection-associated sepsis

Due to their capability of recognizing a variety of PAMPs, TLRs play a critical role in fighting against pathogen infection (Mogensen, 2009). The first identified TLR4 can detect a wide range of Gram-negative bacteria by recognizing and responding to the coating molecules on the bacteria cell wall—lipopolysaccharide (LPS). TLR2 (in conjunction with either TLR1 or TLR6) primarily recognizes lipoproteins/lipopeptides and glycolipids from various pathogens including microbes and fungi. TLR5 is the only TLR that identifies a protein ligand—flagellin from nearly all flagellated bacteria. On the other hand, the endosomal TLRs (TLR3, and TLR7-TLR9) are mainly responsible for the recognition of nucleic acid ligands, including dsRNA, ssRNA, and CpG-DNA, from viral and intracellular bacterial infection. Therefore, activation of TLRs by engaging with these PAMPs allows the host to combat invading pathogens. In fact, TLR agonists have long been considered as vaccine adjuvants to boost the immune system to eliminate viral infections and most recently fight cancer (O'Neill et al., 2009; Hedayat et al., 2011).

However, excessive and uncontrolled TLR activation can lead to overwhelming systemic inflammation and multiple organ injury, characterized as sepsis. Among all TLRs, TLR2, and TLR4 are the two notable contributors to the pathogenesis of sepsis (Tsujimoto et al., 2008). Evidence came from the observation that the expression of both TLR2 and TLR4 was up-regulated during their ligand stimulation in monocytes from healthy subjects (Wittebole et al., 2005), as well as in PBMC from sepsis patients (Harter et al., 2004). In addition, TLR4 knockout mice are resistant to Gram-negative bacteria-induced septic shock (Roger et al., 2009), and TLR2-deficient mice have increased survival rates compared to wild type mice in a polymicrobial sepsis model (Bergt et al., 2013). Moreover, blockade of TLR2 and TLR4 signaling by antagonistic antibodies successfully decreases disease severity in sepsis models of Gram-positive and Gram-negative bacteria, respectively (Meng et al., 2004; Daubeuf et al., 2007). Interestingly, the protective effect can also be seen in the TLR3- and TLR9-deficient mice against microbial challenge; (Cavassani et al., 2008; Hu et al., 2015) and the pharmacological inhibitors of TLR9 can reduce the mortality of mice in severe sepsis and protect the host from serious complications, such as acute kidney injury (Yasuda et al., 2008; Liu et al., 2012). Since sepsis is one of the leading causes of death in the intensive care units worldwide, much effort has been put to develop therapeutic agents blocking TLR activation, especially TLR2 and TLR4, although none has yet been clinically successful (see Section Toll-Like Receptor Antagonists/Inhibitors and Their Clinical Applications).

### Vascular system disorders

Not only can TLRs detect PAMPs in defending pathogen invasion, but they can also recognize circulating host-derived DAMPs released from the dying cells and damaged tissues to trigger the process of self-healing and tissue repair. Till recent years, studies have revealed that sensing the danger signals by TLRs could also contribute to the pathogenesis of many cardiovascular diseases, including atherosclerosis, hypertension, and stroke (Goulopoulou et al., 2016).

Atherosclerosis nowadays is considered as a chronic, progressive inflammatory condition corresponding to lipid accumulation in the vascular system than simply a lipid build-up disease. It has been found that the onset and ongoing of inflammation is mainly caused by the recognition of oxidized lipoproteins (as DAMPs) by TLRs expressed on innate immune cells (e.g., macrophages) and endothelial cells (Miller, 2005); TLR are also involved in the progression of the disease by lesion orientation (Edfeldt et al., 2002), collagen degradation (Monaco et al., 2009), and plaque destruction (Ishibashi et al., 2013). Specifically, it has been shown that deficiency in TLR2 or TLR4 results in diminishing inflammation in the prominent mouse models of atherosclerosis [apolipoprotein E (ApoE)- and LDL receptor-deficient mice; (Michelsen et al., 2004; Mullick et al., 2005, 2008; Higashimori et al., 2011)]. The activation of endosomal TLRs, on the other hand, seems to have controversial effects on atherosclerosis. TLR3 activation has been found to promote atherogenic inflammation, especially in mediating plaque instability (Ishibashi et al., 2013), while the absence of TLR9 exacerbates atherosclerosis in ApoE-deficient mice on a high-fat diet (Koulis et al., 2014).

In the case of hypertension, TLR4 has been well-documented to mediate aberrant immune and inflammation in vasculature, kidneys and autonomic nervous system (McCarthy et al., 2014). This is mainly because TLR4 can respond to angiotensin II, a DAMP, and cause subsequent vascular dysfunction and high blood pressure (Bomfim et al., 2015; Hernanz et al., 2015). Surprisingly, the evidence of TLR2 involved in hypertension is limited to inflammation in the renal system, where TLR2-activated NF-κB signaling is significantly correlated with renal ischemia/reperfusion injury (Khan et al., 2012). Recently, attentions have been drawn to TLR9 for its role in hypertension and blood pressure regulation. Studies have shown that TLR9 activation could elevate blood pressure in normotensive rats (McCarthy et al., 2015), and could also serve as a negative modulator of cardiac autonomic tone and baroreflex function (Rodrigues et al., 2015).

TLR2, TLR4, and endosomal TLRs have all been found to play an important role in stroke and cerebrovascular injury. Interestingly, most of them have a dual function along the time course of the disease: activation of TLRs in post-ischemia state would mediate neuroinflammation and neurodegeneration, but preconditioning with TLR stimulation could be neuroprotective from hypoxia and nutrient deprivation (Wang et al., 2011; Gesuete et al., 2014). For example, TLR7 and TLR8 expression is correlated to the aggravated neuroinflammation and poor outcome in acute ischemic stroke patients (Brea et al., 2011). On the other hand, TLR7 preconditioning [i.e., pre-treatment with a TLR7 agonist, gardiquimod (GDQ), prior to ischemia] has shown protection against subsequent stroke injury through type I IFN-mediated mechanism (Leung et al., 2012).

### Other inflammatory diseases

Activation of TLRs is also involved in many other inflammation-associated diseases, such as psoriasis, gastrointestinal malignancies, and inflammatory airway diseases. Their relationships are briefly described hereafter.

Psoriasis is a long-lasting, relapsing inflammatory disease of dermis and epidermis. It has been observed that the expression of transforming growth factor (TGF)-α and heat shock proteins (e.g., HSP60) from keratinocytes can upregulate TLR5 and TLR9 (Miller et al., 2005), and activate TLR2 and TLR4, respectively (Seung et al., 2007). In addition, activation of TLR7/8 by its specific agonist imiquimod could also aggravate the disease symptoms (Gilliet et al., 2004).

It is known nowadays that chronic inflammation is often linked to carcinogenesis. In particular, chronic gastritis due to *Helicobacer pylori* infection and severe colitis of patients with inflammatory bowel diseases (IBD) greatly increase the risk of having gastrointestinal malignancies (Itzkowitz and Yio, 2004; Houghton and Wang, 2005), and TLR signaling is evidently involved in such inflammatory complications (Fukata and Abreu, 2008). It has been found that TLR4/MyD88 pathway can promote the development of colitis-associated colorectal tumors (Fukata et al., 2007). Since tumor cells also express TLR4, TLR4-induced uncontrolled production of immunosuppressive cytokines has been suggested to contribute to the tumor progression (Sato et al., 2009).

In inflammatory airway diseases, such as asthma and COPD, TLR signaling pathways are closely linked to the disease pathophysiology (Bezemer et al., 2012). For examples, house dust mites, one of the main risk factors for allergic asthma has been suggested to trigger airway neutrophils and monocytes through a TLR4-dependent mechanism (Li et al., 2010). Furthermore, the TLR4 agonist LPS is able to induce either Th1 or Th2 immune response in asthma in a dose dependent manner (Dong et al., 2009). In the case of COPD, TLR2, TLR4, and TLR9 have been shown to participate in cigarette smoke-induced inflammatory responses (Droemann et al., 2005; Karimi et al., 2006; Mortaz et al., 2010). During the exacerbation of the airway diseases, overactive TLR signaling can contribute to excessive inflammatory responses and lung tissue destruction (Tokairin et al., 2008; Stowell et al., 2009).

## Toll-like receptor antagonists/inhibitors and their clinical applications

**Figure 2:**
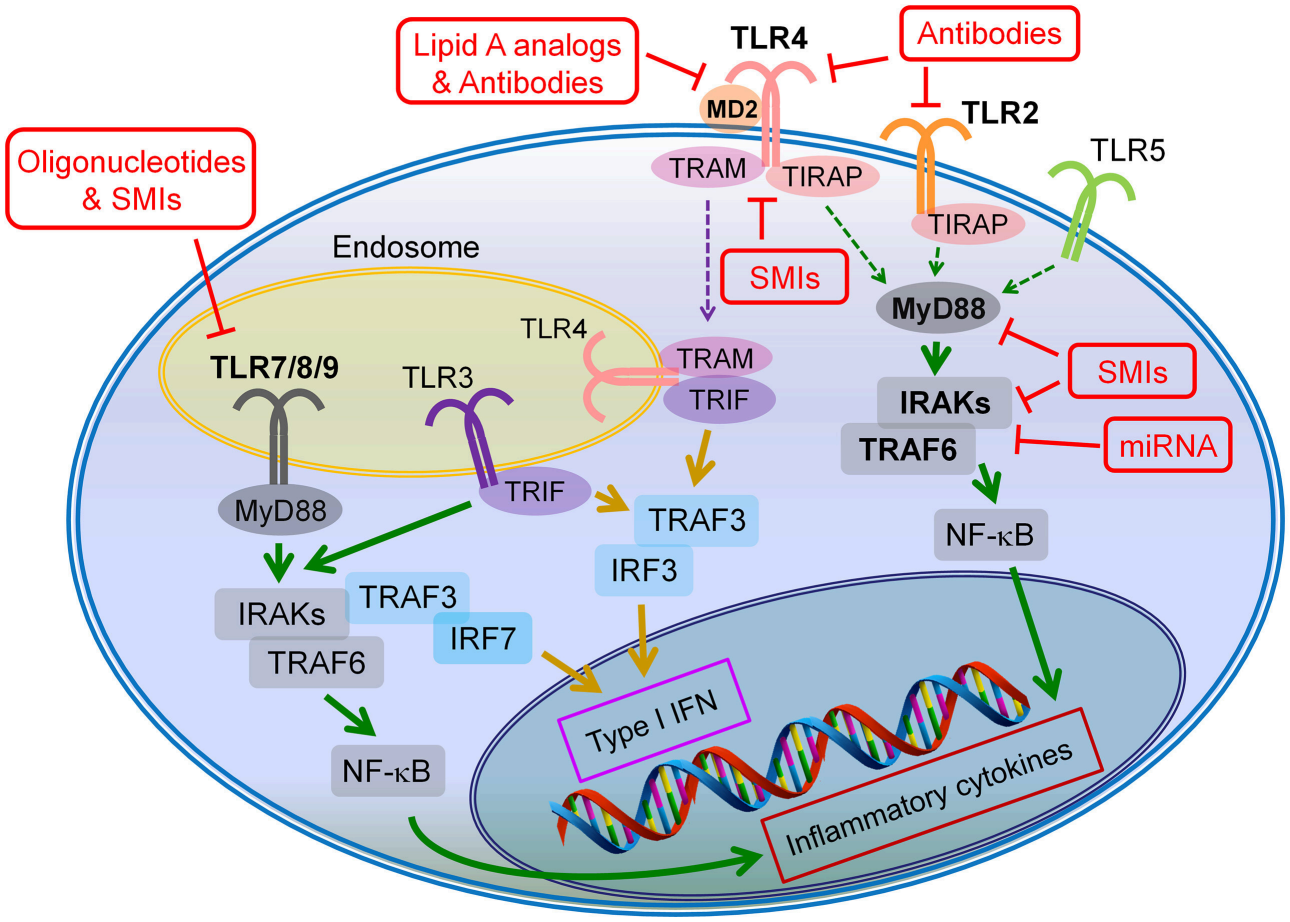
Potential drug targets of TLR signaling pathways. The TLR inhibition can be achieved through two major strategies: (1) blocking the binding of the agonists to the corresponding TLRs, and (2) inhibiting the intracellular signaling of the TLR pathways. The antibodies, lipid A analogs and oligonucleotides primarily target at the ligand-receptor binding, whereas the microRNAs (miRNAs) mainly act on the intracellular signaling cascades of TLR pathways; the small molecule inhibitors (SMIs) can inhibitor TLR signaling through both strategies.

Although, evolutionarily TLRs are essential elements in innate immune system and play a critical role in the host defensive mechanism against invading pathogens, overactivation of TLRs is inevitably involved in the pathogenesis of many inflammatory diseases. Thus, inhibition of TLR signaling pathways has been predicted to be an effective therapeutic strategy to suppress unwanted, disease-associated inflammatory responses. In general, TLR inhibition can be achieved by two major strategies: (1) blocking the binding of TLR ligands to the receptor; (2) interfering the intracellular signaling pathways to stop the signal transduction (Figure 2). Accordingly, various therapeutic agents for inhibiting TLR signaling have been developed to control excessive inflammation; they can be classified as small molecule inhibitors, antibodies, oligonucleotides, lipid-A analogs, microRNAs, and new emerging nano-inhibitors. The current advances of each class are summarized and discussed hereafter (Tables 2–**5**).

**Table 2:** The development status of TLR antagonists/inhibitors: class of SMIs.

**Compound**	**Target**	**Indication**	**Status**	**Company**	**References**
TAK-242	TLR4	Septic shock	Phase III^a^	Takeda	Rice et al., 2010; Matsunaga et al., 2011
Candesartan^1^	ARB, TLR2/4	Anti-inflammation	Animal study	–	Dasu et al., 2009
Valsartan^1^	ARB, TLR4	Myocardial IR injury	Animal study	Novartis Pharma	Yang et al., 2009
Fluvastatin^1^	TLR4	Chronic heart failure	Experimental	–	Foldes et al., 2008
Simvastatin^1^	TLR4	Atherosclerosis	Experimental	–	Methe et al., 2005
Atorvastatin^1^	TLR4	Atherosclerosis	Animal study	–	Fang et al., 2014
ST2825	MyD88	SLE	Experimental	–	Loiarro et al., 2007; Capolunghi et al., 2010
CQ, HCQ	TLR7/8/9	RA, SLE	Clinical use	Generic	Wozniacka et al., 2006; Lesiak et al., 2010; Lee et al., 2011
CQ, HCQ	TLR7/8/9	Cerebrovascular ischemia, lupus-associated hypertension, endothelial dysfunction	Animal study	–	Cui et al., 2013; Gomez-Guzman et al., 2014
CQ	TLR7/8/9	Chikungunya infection	Phase III^a^	Generic	Paton et al., 2011; Borges et al., 2013
		Prevention of influenza	Phase II^b^		
		Dengue infection	Phase I/II^b^		
CpG-52364	TLR7/8/9	RA, SLE	Phase I^c^	Pfizer	Lipford et al., 2007
SM934	TLR7/9	SLE, autoimmune diseases	IND filed^1^	Shanghai Institute of Materia Medica	Hou et al., 2011; Wu et al., 2016

*ARB, angiotensin II receptor blocker; CQ, chloroquine; HCQ, hydroxychloroquine; RA, rheumatoid arthritis; SLE, systemic lupus erythematosus; IND, investigational new drug application*.

a *Study terminated*.

b *Unknown status*.

c *Completed with unknown results*.

1 *Clinical drugs with new applications*.

### Small molecule inhibitors (SMIs)

Small molecule inhibitors (SMIs) are synthetic or naturally derived chemical agents with the activity to inhibit TLR signal transduction. Their amphipathic property and small size allow them to cross cell membranes and act on specific intracellular adapter proteins or compartments along the TLR signaling pathways. They usually have good bioavailability but poor specificity and targeting capability. The ease of manufacturing and handling of SMIs makes them popular drug candidates in pharmaceutical industries (Table 2). In the clinical practice, a group of antimalarial drugs, including hydroxychloroquine sulfate (HCQ), chloroquine (CQ), and quinacrine (or mepacrine), have been used to treat autoimmune diseases (arthritis and SLE) after World War II (Fox, 1996; Ruiz-Irastorza et al., 2010). It was not until recent years that their additional mechanisms of action on endosomal TLR signaling (TLR7/8/9) was identified (Kuznik et al., 2011; Lee et al., 2011). These SMIs are all weak bases and tend to accumulate in the acidic intracellular compartments like endosomes and lysosomes, and are able to modulate the pH in these vesicles. The pH modulation can lead to suppression of autoantigen presentation, blockade of endosomal TLR signaling, and decrease in cytokine production (Fox, 1996; Wozniacka et al., 2006; Kuznik et al., 2011). Other mechanisms include inhibition of MAPK signaling and phospholipase A2, antiproliferation, photoprotection as well as reduction of matrix metalloproteinase-9 (MMP-9) activity (Loffler et al., 1985; Weber et al., 2002; Kim et al., 2006; Wozniacka et al., 2008; Lesiak et al., 2010). These mechanisms of action altogether suggest their anti-inflammatory and immuno-suppressive activity.

In addition to autoimmune diseases, these SMIs of TLR7/8/9 are also under investigation in other diseases associated with uncontrolled acute or chronic inflammation. For example, the antimalarial drug CQ has shown its clinical potential in treating severe sepsis, especially in reducing the risk of sepsis-induced acute renal failure (Yasuda et al., 2008). For its capability of blocking endosomal acidification and anti-inflammation, CQ has been applied to prevent and treat various viral infections, such as HIV, influenza, and dengue (Paton et al., 2011; Borges et al., 2013). In pre-clinical studies, CQ pretreatment can significantly improve the cerebral ischemia symptoms in a transient global cerebral ischemia rat model, which suggests that these SMIs of endosomal TLRs could be beneficial for patients with cardiovascular diseases (Cui et al., 2013). Moreover, long-term administration of HCQ has been found to ameliorate hypertension and aortic endothelial dysfunction in a lupus animal model (Gomez-Guzman et al., 2014). These evidences highlight the great potential of the early developed antimalarial drugs and their derivatives as endosomal TLR SMIs in treating a wide range of inflammatory and autoimmune diseases.

Following the success of these early antimalarial drugs, new SMIs have been developed to target endosomal TLR7/8/9 signaling. Among these newly developments, CpG-52364 is a quinacrine derivative, a SMI of TLR7/8/9 (Lipford et al., 2007). Comparing to HCQ, CpG-52364 is more therapeutically effective and has less side effects from animal studies; a phase I clinical trial was completed in 2009 for SLE treatment (Pfizer: NCT00547014), but the results have not been released since. Another exciting advancement is SM934, a novel analog of artemisinin (or “qinghao su,” the new antimalarial drug identified in the early-70's in China), targeting TLR7/9. Studies have shown the therapeutic effects of the orally administered SM934 on lupus-prone MRL/lpr mice by inhibiting Th1 and Th17 cell responses as well as suppressing the B cell activation and plasma cell production (Hou et al., 2011; Wu et al., 2016). ST2825 is a peptidomimetic compound with an inhibitory activity on the MyD88 dimerization and recruitment of interleukin-1 receptor-associated kinase 1 (IRAK1) and IRAK4 (Loiarro et al., 2007); it has been found to suppress TLR9-induced B cell proliferation and differentiation into plasma cells as well as autoantibody production (Capolunghi et al., 2010). Although ST2825 is primarily used as an experimental agent, it provides evidence of inhibiting MyD88 (the universal adaptor used by most TLRs except TLR3) as a potential therapeutic target to treat patients with SLE and possible other autoimmune diseases.

In addition to endosomal TLR signaling, TLR2 and TLR4 are other two major targets of therapeutic SMIs. TAK-242 (Resatorvid) is an anti-sepsis SMI that targets TLR4 signaling pathways (Matsunaga et al., 2011). This compound binds to cysteine 747 in the intracellular TIR domain of TLR4, which blocks the interaction between TLR4 and the adaptor proteins-TIR domain containing adaptor protein (TIRAP) and TRIF-related adaptor molecule (TRAM), thereby diminishing LPS-induced TLR4 signaling and inflammation. Based on its pre-clinical success, TAK-242 advanced into clinical investigations. Two phase III clinical trials NCT00143611 for severe sepsis and NCT00633477 for sepsis-induced cardiovascular and respiratory failure were launched. In the first trial, the results were unfortunately not satisfactory due to failure to effectively suppress serum cytokine levels (IL-6, IL-8, and TNFα) comparing to controls, even though the drug was well-tolerated (Rice et al., 2010). The second trial, however, was terminated due to a business decision, and no further clinical development of TAK-242 has been conducted ever since.

Like antimalarial drugs, angiotensin II receptor blockers (ARBs), and statins are among the early developed drugs with newly discovered inhibitory activity on TLR2 and TLR4 signaling. For example, the ARB family valsartan can decrease proinflammatory cytokines release and infarct size by inhibiting TLR4 signaling (Yang et al., 2009) while candesartan can suppress Pam3CSK4 and LPS induced TLR2 and TLR4 activation, respectively (Dasu et al., 2009). Statins, also known as 3-hydroxy-3-methyl-glutaryl-coenzyme A (HMG-CoA) reductase inhibitors, are a class of lipid-lowering drugs. Among the statin family, fluvastatin, simvastatin, and atorvastatin all have shown potent inhibitory activity on TLR4 and subsequent inflammatory pathways to reduce inflammation in vascular systems (Methe et al., 2005; Foldes et al., 2008; Fang et al., 2014).

### Antibodies

Due to their high specificity, antibodies have been widely used as biological tools to precisely probe certain molecules. For therapeutic applications, they can be designed to neutralize soluble effectors, block the binding of receptors to their ligands to stop signal transduction, or induce targeted cytotoxicity (Suzuki et al., 2015). The most attractive feature of therapeutic antibodies is their superior specificity to the drug targets comparing to other type of drugs, and hence they are getting great attentions especially for immunotherapy nowadays. However, antibodies still possess a number of limitations including high costs in manufacturing, poor cellular and tissue penetration, and risks of immunogenicity (Chames et al., 2009).

In terms of inhibiting TLR signaling, antibodies are designed (as antagonists) to block the binding of ligands to the specific TLRs (Table 3). One promising advancement is OPN-305, which is the first fully humanized IgG4 monoclonal TLR2-specific antibody. Pre-clinical studies have shown the potency of this compound in blocking TLR2-mediated pro-inflammatory cytokine production *in vitro* and in ischemia-reperfusion (IR) injury animal models (Ultaigh et al., 2011; Arslan et al., 2012; Farrar et al., 2012). The success of OPN-305 in phase I study suggests that OPN-305 is an effective and well-tolerated treatment for the prevention of IR injury in solid organ transplantation (Reilly et al., 2013). It is currently under a phase II clinical trial (NCT01794663) in renal transplant patients with a high risk of developing delayed graft function.

**Table 3:** The development status of TLR antagonists/inhibitors: class of antibodies.

**Compound**	**Target**	**Indication**	**Status**	**Company**	**References**
OPN-305	TLR2	Delayed graft function	Phase II	Opsona therapeutics	Arslan et al., 2012; Reilly et al., 2013
T2.5	TLR2	Septic shock, cardiac fibrosis, cerebrovascular ischemia	Animal study	–	Meng et al., 2004; Spiller et al., 2008; Ziegler et al., 2011; Wang et al., 2014
NI-0101	TLR4	RA	Phase I	NovImmune	Monnet et al., 2015, 2017
1A6	TLR4/MD2	Sepsis	Experimental	–	Spiller et al., 2008; Lima et al., 2015

*RA, rheumatoid arthritis*.

T2.5 is another anti-TLR2 antibody with therapeutic potential. It was found to increase animal survival and protect against severe shock-like syndrome in a mouse model challenged with Pam3CSK4 (a TLR1/2 ligand) or lethally challenged with *Bacillus subtilis* (Meng et al., 2004). A combination therapy of T2.5 with 1A6 (an anti-TLR4 antibody, see below) have shown beneficial effects in a sepsis mouse model challenged by *S. enterica* and *E. coli* (Spiller et al., 2008). In addition to sepsis models, T2.5 treatment was able to decrease neural death and inflammatory responses in a model of transient brain ischemia (Ziegler et al., 2011). Moreover, T2.5 was found to prevent angiotensin II-induced cardiac fibrosis through suppressing macrophage recruitment and inflammation in the heart (Wang et al., 2014). Altogether, these proof-of-principle studies suggest that blocking TLR2 signaling is therapeutically beneficial to sepsis and vascular system disorders. However, this compound was not moved forward into clinical development but remains as a widely used experimental tool now.

NI-0101 is the first monoclonal anti-TLR4 antibody entering clinical development. It can potently inhibit TLR4 signaling by blocking TLR4 dimerization, which is independent on the ligand type and concentration (Monnet et al., 2015). NI-0101 just completed a phase I clinical trial (NCT01808469) and the results were very encouraging (Monnet et al., 2017). NI-0101 was well-tolerated without safety concerns at various doses; it could successfully block cytokine release *ex vivo* and *in vivo*, and prevent flu-like symptoms upon LPS administration. Recent data demonstrated that NI-0101 could also block rheumatoid arthritis (RA) synovial fluids-induced pro-inflammatory cytokine production in monocytes isolated from RA patients (Hatterer et al., 2016). Now, the company is preparing for a phase II clinical trial in RA patients with a TLR4-driven pathotype in 2017.

Another monoclonal antibody of TLR4 is 1A6, which targets the extracellular portion of the TLR4-myeloid differentiation factor 2 (MD2, a co-receptor for TLR4 activation) complex (Spiller et al., 2008). In preclinical study, 1A6 showed protective effect on microbial-induced septic shock *in vivo* (Spiller et al., 2008; Lima et al., 2015). Interestingly, in a mouse model of dextran sulfate sodium (DSS)-induced colitis, 1A6 could ameliorate inflammation and prevent the progression of the intestine inflammation; however, the blockade of TLR4 signaling by 1A6 could also cause a defect in the mucosal healing during the recovery stage (Ungaro et al., 2009). This study perhaps sent a warning message of blocking early TLR4 signaling as potential therapy for inflammatory bowel diseases (IBD); on the other hand, it provided a strong evidence of multiple roles of TLRs in disease development, progression and remission, guiding the development of next generation of therapeutic TLR inhibitors.

### Oligonucleotides

Since endosomal TLRs primarily recognize nucleic acid structures from dsRNA, ssRNA, and CpG-DNA, oligonucleotides with certain sequences are predicted to function as antagonists of endosomal TLRs. They can interfere the binding of endosomal TLRs to their ligands, and hence block the TLR signal transduction. Accordingly, several oligonucleotide-based TLR antagonists have been developed particularly to treat inflammatory diseases associated with endosomal TLR activation, such as SLE; each is discussed hereafter (Table 4).

**Table 4:** The development status of TLR antagonists/inhibitors: class of oligonucleotides.

**Compound**	**Target**	**Indication**	**Status**	**Company**	**References**
IRS-954	TLR7/9	SLE	Preclinical^a^	Dynayax Technologies	Barrat et al., 2005, 2007; Guiducci et al., 2010
DV-1179	TLR7/9	SLE	Phase Ib/IIa^a^	Dynayax Technologies	Zhu et al., 2011; Suarez-Farinas et al., 2013
IMO-3100	TLR7/9	SLE	Preclinical	Idera	Zhu et al., 2011; Suarez-Farinas et al., 2013
		Psoriasis	Phase II^b^		
IMO-8400	TLR7/8/9	Dermatomyositis	Phase II	Idera	Jiang et al., 2012; Zhu et al., 2012
		Plaque psoriasis	Phase II		
IMO-9200	TLR7/8/9	Autoimmune diseases	Phase I	Idera/Vivelix	–
IHN-ODN 2088	TLR9	Hypertension	Animal study	–	McCarthy et al., 2015
IHN-ODN-24888	TLR7/9	SLE	Preclinical	Coley Pharmaceutical GmbH	Rommler et al., 2013, 2015

*SLE, systemic lupus erythematosus*.

a *Study discontinued*.

b *Completed with unknown results*.

One of the leading developments of oligonucleotide-based TLR antagonists is named immunoregulatory DNA sequence (IRS; by Dynavax Technologies). Advancing from the earlier encouraging prototypes, IRS-661 (TLR7 specific) and IRS-869 (TLR9 specific), a dual-function targeting both TLR7 and TLR9 antagonist, IRS-954 was developed for the treatment of SLE (Barrat et al., 2005). IRS-954 was found to inhibit IFN-α production by human plasmacytoid predendritic cells in response to DNA or RNA of viruses and immune complexes from SLE patients. In addition, IRS-954 treatment showed a reduction of autoantibodies and improvement of disease symptoms in lupus-prone mice (Barrat et al., 2007). Furthermore, it could also reverse TLR7/9-mediated glucocorticoid resistance of SLE, and thus could be potentially used as corticosteroid-sparing drug (Guiducci et al., 2010). Although IRS-954 has shown promising pre-clinical therapeutic efficacy, there is no further advancement documented in the clinical development to date. On the other hand, DV-1179, another TLR7/9 dual antagonist developed by the same company, entered a phase Ib/IIa study (in partner with GSK) for its safety in both healthy volunteers and patients with active SLE (Dynavax, 2014). Although DV-1179 administration was well tolerated, it failed to achieve the endpoints of reducing the IFN-α-regulated genes^1^. Such an unfortunate outcome ended the clinical development of this compound ever since.

Another development of oligonucleotide-based antagonists (also termed as immune modulatory oligonucleotide IMO) has made some success into clinical trials (by Idera Pharmaceuticals). The compound IMO-3100 is also a TLR7/9 dual antagonist. In preclinical studies, IMO-3100 could significantly reduce the expression of inflammatory genes, such as IL-17A, β-defensin, CXCL1, and keratin 16 in a mice model of IL-23-induced psoriasis (Suarez-Farinas et al., 2013). Moreover, this compound showed an inhibitory activity on disease progression in a lupus-prone mice model (Zhu et al., 2011). A phase II trial for the treatment of psoriasis was completed in 2012 (NCT01622348), but the results have not yet been disclosed. IMO-8400 is another candidate under current development. Different from IMO-3100, IMO-8400 is capable of inhibiting all TLR7, TLR8, and TLR9. This compound was very effective in preventing inflammation and disease development in both lupus and psoriasis mouse models (Jiang et al., 2012; Zhu et al., 2012; Suarez-Farinas et al., 2013). Currently, two phase II clinical trials are underway for the treatment of plaque psoriasis (NCT01899729) and dermatomyositis (NCT02612857). Similar to IMO-8400, a newly developed IMO-9200 can also target TLR7/8/9. From a phase I clinical trial (in partner with Vivelix), it showed a safe and well-tolerated profile in healthy subjects^2^.

There are other immunoregulatory oligonucleotides under preclinical developments. The inhibitory oligonucleotide (IHN-ODN) 2088 is a potent TLR9 antagonist. In spontaneously hypertensive rats, IHN-ODN 2088 treatment showed positive effects on systolic blood pressure reduction (McCarthy et al., 2015). Based on IHN-ODN 2088, a guanine-modified inhibitory oligonucleotide, INH-ODN-24888, was designed; this compound demonstrated a promising therapeutic effect in SLE through the suppression of both TLR7 and TLR9 (Rommler et al., 2015). The guanine modification potentiates the inhibitory activity of INH-ODN-24888 and efficiently improves its capacity to reduce TLR7/9-mediated immune signaling in human immune cells (Rommler et al., 2013). These results indicates that IHN-ODN-24888 may be further developed into a promising clinical treatment for SLE.

### Lipid A analogs

A special group of TLR antagonists is the lipid A analog (Table 5), specifically targeting TLR4. Lipid A is a lipid component of LPS (or endotoxin) contributing to the toxicity of an endotoxin molecule. Because the structure of lipid A is highly conserved in endotoxins, it becomes an attractive therapeutic target to regulate TLR4 signaling (Rietschel et al., 1994).

**Table 5:** The development status of TLR antagonists/inhibitors: classes of lipid A analogs, miRNAs and “nano-drugs.”

**Compound**	**Target**	**Indication**	**Status**	**Company**	**References**
**CLASS OF LIPID A ANALOGS**					
Eritoran (E5564)	TLR4/MD2	Sepsis	Phase III^a^	Eisai (Boston)	Barochia et al., 2011; Opal et al., 2013
		Influenza infection, cardiac hypertrophy, myocardial IR injury, renal IR injury	Animal study	–	Shimamoto et al., 2006; Liu et al., 2010; Ehrentraut et al., 2011; Shirey et al., 2013
**CLASS OF miRNAs**					
miR-146a	TLR4	Autoimmune diseases, SLE	Animal study	–	Taganov et al., 2006; Tang et al., 2009; Boldin et al., 2011; Pan et al., 2012
miR-21	TLR4	Anti-inflammation	Animal study	–	Sheedy et al., 2010
**CLASS OF “NANO-DRUGS”**					
NAHNP	TLR4	Anti-inflammation	Experimental	–	Babazada et al., 2014a,b
HDL-like NP	TLR4	Anti-inflammation	Experimental	–	Foit and Thaxton, 2016
Bare GNP	TLR4	Eye inflammation	Animal study	–	Pereira et al., 2012
Glycolipid-coated GNP	TLR4/MD2	Sepsis	Experimental	–	Rodriguez Lavado et al., 2014
Peptide-GNP hybrid	TLR2/3/4/5	Anti-inflammation	Experimental	–	Yang et al., 2015, 2016

*SLE. systemic lupus erythematosus; NAHNP, non-anticoagulant heparin nanoparticle; HDL, high-density lipoprotein; NP, nanoparticle; GNP, gold nanoparticle*.

a *Study discontinued*.

A synthetic lipid A analog of *Rhodobacter sphaeroides*, eritoran (E5564), is probably the most exciting but also disappointing clinical development of TLR4 antagonist (by Eisai Research Institute of Boston Inc.) (Barochia et al., 2011). Eritoran is the 2nd generation of its own kind derived from the first developed E5531 (Christ et al., 1995). The design principle of these compounds was to competitively bind to the MD2 pocket by mimicking the lipid A structure, which in turn prevents the LPS binding and the induction of TLR4 signaling (Park et al., 2009). The structural improvement of eritoran makes it superior to E5531 for its higher potency due to extended duration of action, cost-effective manufacturing because of a simpler structure, greater water solubility, and better chemical stability. Preclinically, eritoran could significantly reduce LPS-induced NF-κB activation and pro-inflammatory cytokine production (TNF-α, IL-1β, IL-6, and IL-8) *in vitro* and in animal models (Mullarkey et al., 2003; Savov et al., 2005). In a phase I clinical study on LPS challenged healthy volunteers, eritoran treatment effectively decreased TNF-α and IL-6 levels in the blood, reduced the white blood cell counts and C-reactive protein levels, and diminished sepsis-associate clinical symptoms; the only observed adverse response was the dose-dependent phlebitis (Lynn et al., 2004; Rossignol et al., 2004, 2008). It then went on a randomized, double-blind, multicenter phase II trial (NCT00046072) to treat critically ill septic patients with high predicted risk of mortality; the results were positive as the intravenous treatment of eritoran at the dose of 105 mg led to a trend toward a lower mortality rate (Tidswell et al., 2010). Sadly, the phase III trial (ACCESS, NCT00334828) of eritoran on severe sepsis patients failed because eritoran did not reduce the patient mortality at 28 days and 1 year from the statistical analysis (Opal et al., 2013). Accordingly, the company decided to give up launching the drug to the market.

Although the development of eritoran led to a disappointing end, valuable lessons can be learned from this failed practice for developing the next generation of TLR inhibitors. First, the patient recruitment is key to objectively evaluate the drug efficacy in a clinical trial. One major reason that eritoran failed in the ACCESS was probably because the recruited patients were not well-assessed according to their disease pathogenesis, particularly the circulating levels of LPS in the patients. This is a critical factor that should be included in the recruiting criteria. Since the mechanism of eritoran is to block LPS binding to TLR4, those patients who had low levels of circulating LPS at the time of studies may not respond well to the treatment, and thus contributed to an overall null efficacy of the drug. Second, the time and route of drug administration need to be optimized. Considering the acute inflammatory responses in sepsis, the timing of eritoran administration could be too late to be therapeutic effective when other inflammatory mediators started to take over. Third, the genetic backgrounds, the infection status, and the disease severity all contributed to the heterogeneity in the patients, which ultimately resulted in different responses toward the treatment. Finally, the inflammatory responses of the septic patients could be triggered by multiple TLR pathways, and blocking one of them (even though TLR4 has been considered the major one) may not be enough to stop the overwhelming inflammation in severe sepsis. To ensure future success in developing new TLR inhibitors for inflammatory diseases like sepsis, perhaps new inhibitory strategy on multiple TLR pathways should be considered; and clearly in the clinical trials, patients should be carefully selected and examined based on appropriate criteria with well-defined biomarkers to obtain the fair evaluation and interpretation of the therapeutic efficacy of the tested drugs.

Apart from treating sepsis, eritoran could still be therapeutically beneficial for other inflammatory diseases. In fact, one study has shown that eritoran could prevent influenza-induced death in a mouse model, where it improved the clinical symptoms, inhibited cytokine production (TNF-α, IL-1β, CXCL1, and IL-6), and reduced viral titers (Shirey et al., 2013). Other studies have shown the therapeutic efficacy of eritoran in several cardiovascular disease models. For example, eritoran could reduce cardiac hypertrophy in an aortic constriction mouse model (Ehrentraut et al., 2011), and attenuate inflammatory cytokine production and myocardial IR injury in a rat model (Shimamoto et al., 2006). In addition, eritoran has also provided protective effects on kidney IR injury (Liu et al., 2010). Unexpectedly, it has also been suggested that eritoran could potentially overcome the blood-brain barrier under certain inflamed conditions (like stoke) and play an advantageous role in treating cerebral infarction (Buchanan et al., 2010).

### miRNA inhibitors

MicroRNAs (miRNAs) are small (~22 nucleotides), endogenous non-coding RNAs with post-transcriptional regulatory functions to finely tune gene expression (Bartel, 2009). They bind to a target sequence in the 3′-untranslated region (UTR) of the messenger RNA (mRNA) to facilitate mRNA degradation or inhibit its translation. Since miRNAs are involved in the regulation of diverse biological processes and associated with pathogenesis of many diseases, they have emerged as new therapeutic targets.

Till now, there are about 20 miRNAs identified to be involved in the regulation of TLR signaling pathways (He et al., 2014). Among them, miR-146a, miR-155, and miR-21 are the three miRNAs received extensive attention for their regulatory roles in TLR signaling and autoimmune diseases (Table 5; Quinn and O'Neill, 2011; Shen et al., 2012). MiR-146a was identified from a screen of LPS-responsive genes regulated by NF-κB; it was found to inhibit the translation of the TNF receptor-associated family 6 (TRAF6) and IRAK1, both of which are in the downstream pathway of TLR4 signaling (Taganov et al., 2006). Further studies have shown that miR-146a could play a negative regulatory role in autoimmunity (Boldin et al., 2011), particularly in blocking the type I IFN production in human lupus by targeting TLR-type I IFN associated genes TRAF6, IRAK1, IFN regulatory factor 5 (IRF5), and signal transducer and activator of transcription 1 (STAT1; Tang et al., 2009). Given the fact that miR-146a acts on multiple targets in the signaling cascades of TLR-mediated induction of type I IFN, the integrated effects of negative regulation by miR-146a on the pathway are expected to be beneficial in treating SLE and perhaps RA. To translate miR-146a into clinic uses, one possible way is to apply a delivery vehicle to help miR-146a cross the cell membranes in order to function intracellularly (Pan et al., 2012).

Both miR-21 and miR-155 were originally identified as “onco-miR,” but later discovered to have a new role in regulating TLR signaling and inflammation. They were found to be upregulated in TLR-associated inflammatory conditions (O'Connell et al., 2007; Sheedy et al., 2010). Specifically, miR-21 could enhance the LPS-induced anti-inflammatory cytokine IL-10 production by lowering programed cell death 4 gene (*PDCD4*) expression, serving as a negative regulator of the inflammatory response (Sheedy et al., 2010). MiR-155, on the other hand, appears to have controversial roles in controlling inflammation. In one study, miR-155 targeted TGF-β activated kinase 1 (TAK1) binding protein 2 (TAB2) and inhibited TAK1 activation, which subsequently blocked the activation of downstream NF-κB and MAPK pathways, and dampened the inflammatory responses (Ceppi et al., 2009). Another study reported that miR-155 expression contributed to the production of the proinflammatory cytokine TNF-α and increased the susceptibility to septic shock (Tili et al., 2007). In addition, miR-155 was found to have a positive effect in the pathogenesis of SLE, as miR-155 knockout mice had phenotypes similar to some human systemic autoimmune disorders, and were highly resistant to experimental autoimmune encephalomyelitis, a SLE mouse models (O'Connell et al., 2010). Accordingly, a miR-155 antagonist, MRG-107, is currently under preclinical development (by miRagen Therapeutics) to treat neuro-inflammation.

### New emerging nano-inhibitors

**Figure 3:**
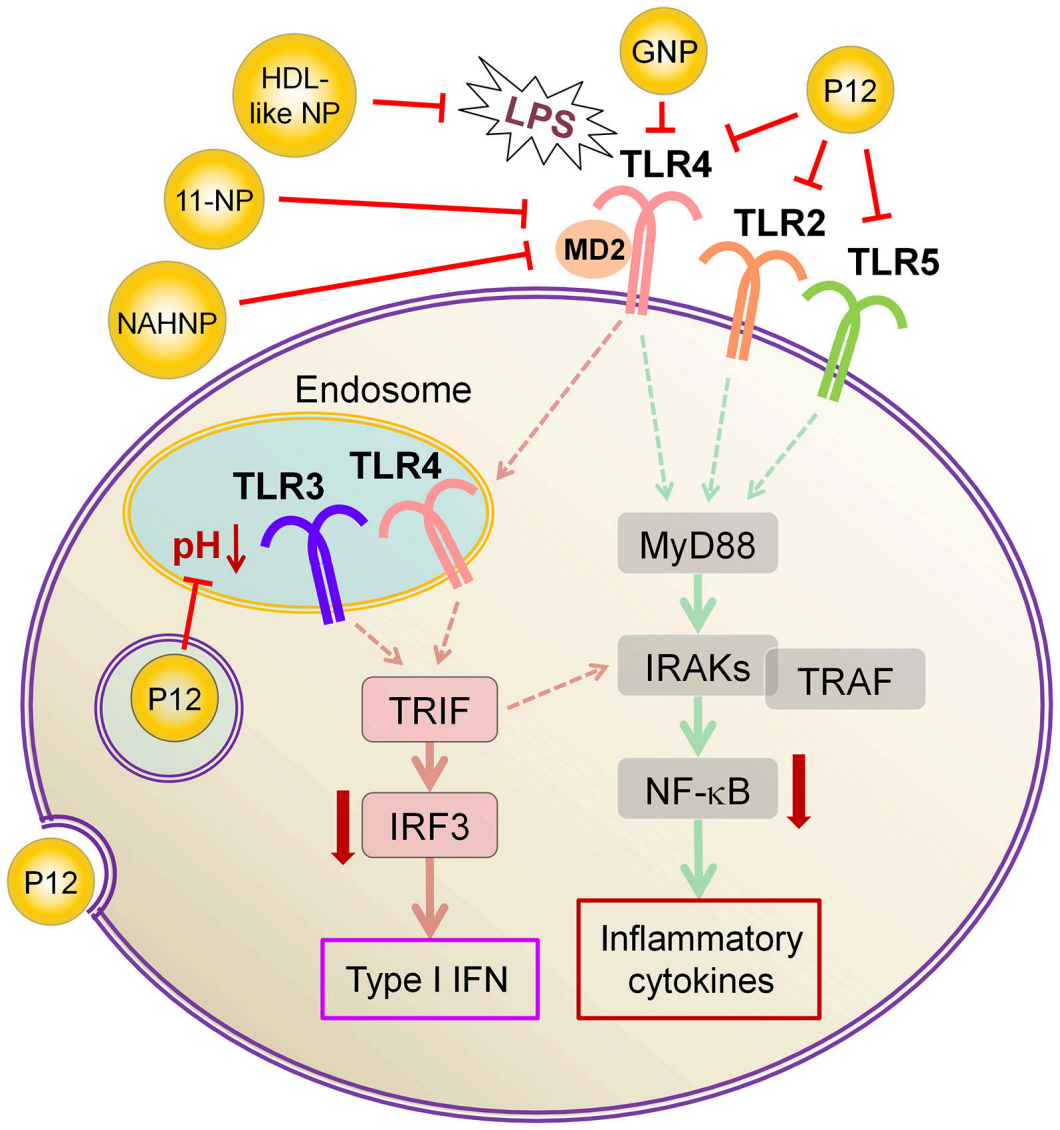
The mechanisms of action of emerging nano-inhibitors targeting TLR signaling pathways. The HDL-like NP is capable of sequestering LPS to down-regulate TLR4 signaling; NAHNP and 11-NP can block the LPS binding to TLR4-MD2 complex, and thus inhibit TLR4 signal transduction; bare gold nanoparticle (GNP) is able to down-regulate TLR4 expression; the peptide-gold nanoparticle hybrid P12 can inhibit multiple TLR pathways including TLR2, 3, 4 and 5, and block the endosomal acidification. These nano-inhibitors can eventually down-regulate the transcription factors NF-κB and IRF activation, leading to the inhibition of the production of inflammatory cytokines and type I interferons.

In addition to above described inhibitors/antagonists, nanodevices are emerging as a new class of potent TLR inhibitors that can target a single or multiple TLR pathways (Table 5). Due to their nanoscale sizes, nano-inhibitors are expected to have better bio-distribution and sustained circulation (He et al., 2010; Blanco et al., 2015). They can be functionalized to meet the desired pharmacodynamic and pharmacokinetic profiles (Ernsting et al., 2013; Lin and Tam, 2015). One distinct feature of these new nano-inhibitors is that the bio-activity comes from their self-properties, which can be tailored to different medical complications, rather from a carried therapeutic agent. This characteristic makes them a special class of drug (or “nano-drug”) as the next generation nano-therapeutics. Currently, they are at the preclinical investigation stage. Figure 3 summarizes their targets in the TLR pathways, whereas their structures, physicochemical properties and mechanisms of action are listed in Table 6.

**Table 6:** The structures, physicochemical properties, and mechanisms of the novel TLR nano-inhibitors.

**Compound**	**NAHNP**	**HDL-like NP**	**Bare GNP**	**Glycolipid-coated GNP**	**Peptide-GNP hybrid**
**Structure**	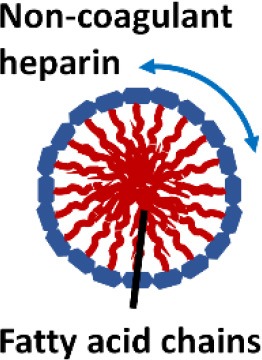	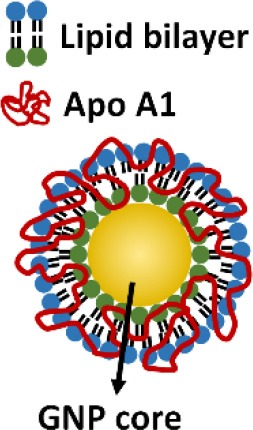	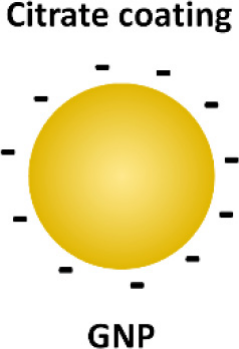	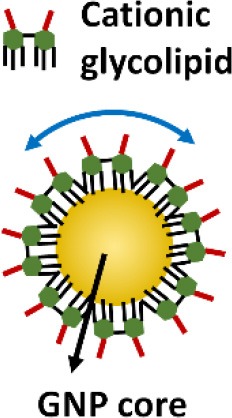	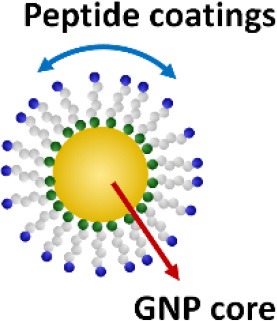
**Size**	110–160 nm	~15 nm	~30 nm	N/A	~15 nm
**NP formation**	Self-assembling	Surface coating on GNP	GNP only (citrate coated)	Surface coating on GNP	Surface coating on GNP
**Surface charge**	Negative	Negative	Negative	Positive	Negative
**Biodegradability**	Possible	No	No	No	No
**Mechanisms of action**	Interaction with TLR4/MD2 complex	Neutralization of LPS	Decrease in TLR4 and NF-κB expression	Binding to TLR/MD2 complex	Endosomal pH modulation
	Inhibiting MyD88-dependent NF-κB pathway	Inhibiting LPS induced NF-κB signaling		Inhibiting LPS induced NF-κB signaling	Inhibiting both NF-κB and IRF pathways
**References**	Babazada et al., 2014a,b	Foit and Thaxton, 2016	Pereira et al., 2012	Rodriguez Lavado et al., 2014	Yang et al., 2015, 2016

*NAHNP, non-anticoagulant heparin nanoparticle; HDL, high-density lipoprotein; NP, nanoparticle; GNP, gold nanoparticle; Apo A1, apolipoprotein A1; N/A, not available*.

One interesting immunomodulatory nanodevice is the lipid-conjugated non-anticoagulant heparin nanoparticle (NAHNP; Babazada et al., 2014a,b). In addition to its well-known anti-coagulant function, heparin is known to have anti-inflammatory activity for its ability to bind and inhibit various cytokines, growth factors and enzymes that are involved in inflammation (Tyrrell et al., 1999); especially, the 6-O sulfation of glucosamine residues of heparin can block the binding of P- and L-selectins for the recruitment of leukocytes at inflammatory sites (Wang et al., 2002). Being intrigued by the intrinsic anti-inflammatory properties of heparin, Babazada et al. synthesized the glycol-split non-anticoagulant heparin with D-erythro-sphingosine grafts, which can self-assemble into NAHNP (Babazada et al., 2014b). These nanoparticles (NPs) were found to suppress LPS-induced MyD88-dependent NF-κB activation and inhibit subsequent cytokines production in mouse macrophages. They further discovered that the length of the alkyl chains of conjugated D-erythro-sphingosine was critical for the anti-inflammatory activity as shortening the alkyl chain of NAHNP resulted in loss of activity. Again, the 6-O-sulfate groups of d-glucosamine residue were important for such an inhibitory activity. By removing the anti-coagulant activity of heparin, NAHNP could serve as new anti-inflammatory nano-therapeutics.

Another novel nanodevice, the high-density lipoprotein (HDL)-like nanoparticle (HDL-like NP), was developed as a TLR4 antagonist by sequestering LPS (Foit and Thaxton, 2016). HDL has been known to naturally bind to and neutralize LPS (Brandenburg et al., 2002), and has been applied to reduce LPS-induced inflammation *in vivo* (Pajkrt et al., 1996; Guo et al., 2013). The HDL-like NP was designed with a gold NP core and a HDL coating, where the lipid components of HDL can be modified (Foit and Thaxton, 2016). Through screening five variants of the lipid coatings, one HDL-like NP was identified to be able to potently inhibit TLR4 signaling triggered by various sources of LPS and Gram-negative bacteria on human cell lines and PBMC. As expected, the acting mechanism of this HDL-like NP was through scavenging the LPS molecules. It is worth noting that this newly synthesized HDL-like NP exhibits much higher anti-inflammatory activity than the HDL alone, making it a promising candidate as a next generation nano-antagonist of TLR4.

Gold nanoparticles (GNPs) have caught much attention as emerging nanotheranostics in nanomedicine, owing to their ease of fabrication and functionalization, chemical stability, excellent photothermal physical properties, and good biocompatibility (Pedrosa et al., 2015). The fact that a TNF-α-bound GNP agent (CYT-6091 by CytImmune Sciences) showed very-well tolerated profile in a phase I clinical trial warrants the potential clinical uses of GNP-based therapeutics in the near future (Libutti et al., 2010). The exciting advancement in the field is the development of GNP-based “nano-inhibitors” for TLR signaling. In one study, bare GNP was applied as the post-treatment topically to ameliorate LPS-induced eye inflammatory responses and reduce the oxidative damage in irides (Pereira et al., 2012). The possible mechanism was through down-regulating TLR4 expression and the corresponding LPS-induced NF-κB activation. This discovery is quite surprising as the bare GNP is physiologically unstable, and is usually thought to be inert without biological activity. It would be interesting to see the follow-up study and the detailed working mechanisms of bare GNP. In another study, a cationic glycolipid-coated GNP system was developed as a new TLR4 antagonist (Rodriguez Lavado et al., 2014). A new class of glycolipids was synthesized to have a structure mimicking lipid A binding to CD14 and the TLR-MD2 pocket. The biological analysis showed that the GNP coated with a specific glycolipid (11-NP) was able to inhibit the LPS-induced TLR4-MD2 activation in human and murine cells; such an effect was similar to the very efficient TLR4 antagonist eritoran. Further studies on the structure-activity relationship analysis indicated that the presence of fatty ester chains and the facial amphiphilicity of the glycolipids are required to maintain TLR4 antagonistic activity. Preliminary *in vivo* studies showed that 11-NP was very effective in reducing TNF-α level in the blood in a LPS mouse model.

One exciting invention is the development of a completely novel peptide-GNP hybrid system as a next generation nano-inhibitor led by Yang et al. (2015, 2016). The system is made of a GNP core coated with a hexapeptide layer to enhance the physiological stability of the GNP, change the surface physicochemical characteristics, and enable the biological activity (Yang et al., 2011, 2013). Through, screening a small library of the established, physiologically stable peptide-GNP hybrids, they discovered a potent multi-TLRs inhibitor, P12, which not only suppressed both arms of TLR4 signaling (i.e., MyD88-dependent NF-κB and TRIF-dependent IRF3 activation), but also inhibited TLR2, TLR3, and TLR5 pathways. The structure-activity relationship analysis revealed that the activity index is strongly dependent on the surface properties of the hybrid, where the hydrophobicity and the aromatic ring-like structure are the two key factors dictating the inhibitory activity (Yang et al., 2015). Further studies conferred the anti-inflammatory activity of P12 in correcting LPS-induced gene expressions, reducing the subsequent pro-inflammatory cytokine production (IL-12p40, MCP-1, and IFN-γ), and increasing the anti-inflammatory cytokine IL-1RA. In searching for the mechanism of action, delicate experiments were performed to show that the potent inhibitory activity of P12 is mainly due to its endosomal pH modulation capability (Yang et al., 2016). Like chloroquine (CQ), a lysotropic agent that can elevate endosome/lysosome pH, P12 was able to prevent the endosomal acidification process, which in turn attenuated downstream signal transduction of endosomal-dependent TLR signaling. More importantly, the inhibitory activity of the “nano-drug” can be well-tuned by selecting different amino acids that are displayed on the GNPs. The *in vivo* efficacy of P12 was investigated using a murine model of intestinal inflammation; it was found that P12 treatment could reduce the animal weight loss, improve the disease activity index, and ameliorate colonic inflammation.

All the above nanoparticles have minimum acute toxicity in culture cells as well as in the limited animal studies. However, since the gold nanoparticle is not biodegradable, the long-term safety issue of using GNP-based therapeutics is still a concern, even though they are well-tolerated in a phase I study. Therefore, addressing the long-term safety problem of GNP-based TLR inhibitors is critical in order to move this line of research into clinic uses. Alternatively, one could replace the GNP core with a biodegradable NP while maintaining the inhibitory activity, preferably targeting multiple TLRs like peptide-gold nanoparticle hybrids, to accelerate the translation of these novel agents into clinical treatments for inflammatory diseases.

Note that the majority of these developed TLR nano-inhibitors act on TLR4 signaling, except the peptide-gold nanoparticle hybrid P12 which has shown inhibitory activity on multiple TLRs. In the current literatures reviewed, LPS has been used universally as the TLR4 agonist. However, other purinergic receptors, such as P2X7 receptor (encoded by *P2RX7* gene), can also recognize LPS and induce the secretion of inflammatory mediators in macrophages (Denlinger et al., 2001). Interestingly, the recognition of LPS by P2X7 requires a co-receptor CD14, which facilitates LPS internalization and binding to the intracellular C-terminal domain of P2X7 (Dagvadorj et al., 2015). Therefore, the specificity of these nanoparticles in inhibition of TLR4 vs. other membrane receptors that also recognize LPS needs to be further addressed. It will also be interesting to see whether these nano-inhibitors can also modulate signaling of other TLRs in addition to TLR4.

Currently, these TLR nano-inhibitors are still at the preclinical investigation stage. Their pharmacokinetics and pharmacodynamics profiles in small and large animals need to be accomplished before launching any clinical trials. However, a few nanoparticles/nano-formulations have already been approved by FDA to deliver water-insoluble drugs or biologics, and have shown enhanced efficacy and safety profiles in cancer treatments. Some well-known examples include the polyethylene glycol (PEG) formulated L-asparaginase (Oncaspar by Sigma-Tau Pharmaceuticals, Inc.) and liposome formulated doxorubicin (Doxil by Jansen Pharmaceutical). Furthermore, several inorganic nanoparticles have been approved by FDA as diagnostic imaging agents. For instance, the iron oxide nanoparticles (Feridex by Bayer HealthCare Pharmaceuticals Inc.) have been used in medical magnetic resonance imaging (MRI). Recent records of clinically used nanoparticles are summarized in two comprehensive reviews in 2016 (Anselmo and Mitragotri, 2016; Bobo et al., 2016). Different from the clinically used nano-therapeutics, the reviewed TLR nano-inhibitors herein would represent a new class of “nano-drug” modulating TLR signaling, and hold great promise for clinical use in the future.

## Concluding remarks

TLRs are very important PRRs in the first-line defense system of our bodies. They can recognize both invading pathogens and endogenous danger molecules released from dying cells and damaged tissues, and play a key role in linking innate and adaptive immunity. Despite their well-known function in sensing pathogens and mounting defense mechanisms, overactivation of TLRs can ultimately lead to disruption of immune homeostasis, and thus increase the risk for inflammatory diseases and autoimmune disorders, such as SLE, RA, atherosclerosis, stoke, IR-induced injury, COPD, asthma, gastrointestinal inflammation, and even cancer. Evidences have shown the involvement of TLRs in the development and progression of these diseases. Accordingly, antagonists/inhibitors targeting TLR signaling pathways have emerged as novel therapeutics to treat these diseases. Although, this therapeutic strategy has been well-accepted experimentally, there are only limited numbers of TLR inhibitors/antagonists that have undergone clinical trials to date; many of them failed during the trials, and none have been approved for clinical uses. Lessons have been learned from these failed developments. The efficacy, safety, and stability of the new therapeutic agents, as well as whether these therapies could impair local immune defenses should be considered comprehensively in the clinical development. More importantly, given the facts that pathogen infection or danger signals often activate multiple TLRs, and that the failure of TLR4 specific antagonist/inhibitor in the clinical trials despite its success in preclinical studies, developing more potent antagonists/inhibitors that target multiple TLR signaling pathways perhaps could shine the way to facilitate the translation of such a promising strategy into clinical uses in the future. Certainly, nano-devices will play an important role in the development of next generation TLR inhibitors.

## Author contributions

QL and HY proposed and designed the scope of the review. WG and HY wrote the manuscript. YX and QL critically revised and commented on the manuscript. All the authors read and approved the final manuscript and agreed to be accountable for the accuracy and integrity of any part of the manuscript.

### Conflict of interest statement

The authors declare that the research was conducted in the absence of any commercial or financial relationships that could be construed as a potential conflict of interest.

## Footnotes

^1^http://www.streetinsider.com/Corporate+News/…DV1179…/9729788.html

^2^http://vivelix.com/research-and-development/
